# Follow-Up of Thyroid Function in Children With Neonatal Hyperthyroidism

**DOI:** 10.3389/fendo.2022.877119

**Published:** 2022-05-04

**Authors:** Beata Pyrżak, Małgorzata Rumińska, Ewelina Witkowska-Sędek, Anna Kucharska

**Affiliations:** Department of Paediatrics and Endocrinology, Medical University of Warsaw, Warsaw, Poland

**Keywords:** neonatal hyperthyroidism, central hypothyroidism, maternal TRAb, Graves’ disease, neonates

## Abstract

**Introduction:**

Neonatal hyperthyroidism mainly occurring in the children born to mothers with Graves’ disease (GD). The influence of maternal GD on the newborn’s thyroid function includes not only hyperthyroidism, but also various forms of hypothyroidism. Maternally transferred thyrotropin receptor antibodies (TRAb), the antithyroid drug (ATD) administration during pregnancy and previous definitive treatment of GD (radioactive iodine therapy or thyroidectomy) in the mother impact the function of the fetal/neonatal thyroid. Some newborns born to mothers with GD may present central hypothyroidism (CeH) due to impaired regulation of the fetal hypothalamic-pituitary-thyroid axis. The aim of this study was to evaluate different types of thyroid dysfunction in babies with neonatal hyperthyroidism.

**Materials and Methods:**

Medical records of 14 infants with neonatal hyperthyroidism (13 born to mothers with GD, and one born to mother with Hashimoto thyroiditis) were analyzed.

**Results:**

Transient hyperthyroidism was the main thyroid dysfunction in our study group. Overt hyperthyroidism with highly increased TRAb levels (mean 13.0 ± 7.0 IU/L) was diagnosed in 6 (43%) neonates. Another 6 (43%) babies presented hyperthyroidism with slightly increased fT4 and/or fT3 levels and TSH levels in the lower limit of the normal range coinciding with positive TRAb levels (mean 3.8 ± 1.6 IU/L). Normal thyroid hormone levels with TSH levels below the lower limit of the range were observed in 2 (14%) neonates. Four babies in the study group (28.5%) required further levothyroxine (L-T4) supplementation due to CeH or, in one case, due to primary hypothyroidism.

**Conclusion:**

Our study highlights the need for prolonged monitoring of thyroid function in children born to mothers with GD. Diagnosis of CeH could be delayed due to its masking by transient hyperthyroidism. Prolonged thyroid-stimulating hormone suppression after TRAb elimination should be considered as a signal announcing CeH.

## Introduction

Neonatal hyperthyroidism is a rare disorder, mainly occurring in the children of mothers with Graves’ disease (GD). The influence of maternal GD on the newborn’s thyroid function includes not only hyperthyroidism, but also various forms of hypothyroidism. Maternal GD affects 0.1-0.4% of pregnancies (1–4). The fetal thyroid gland becomes responsive to thyroid-stimulating hormone (TSH) and thyrotropin receptor antibodies (TRAb) at around 20 weeks of gestation (5, 6). In mothers with GD during pregnancy, thyroid autoantibodies levels usually decrease due to immunosuppression and/or hemodilution and reach their lowest values shortly before delivery. In the postpartum period, the levels of thyroid autoantibodies recover in the mother’s blood and may exceed values detected in early pregnancy (7). In pregnancy, thyroid autoantibodies freely cross the placenta and either overstimulate (thyroid stimulating antibody - TSAb) or block (thyroid blocking antibody - TBAb) the fetal thyroid gland (4, 7–9). Maternally transferred antibodies can transiently impact fetal and neonatal thyroid function until they are metabolized. A high level of TSAb transmission is associated with the occurrence of fetal and neonatal thyrotoxicosis. Maternal TBAb can induce congenital hypothyroidism (9). Thyroid function disturbances observed in the fetus/newborn depend not only on the type of maternal antibodies, but also on their levels. The antithyroid drug (ATD) administration during pregnancy and previous definitive treatment of GD (radioactive iodine [RAI] therapy or thyroidectomy) in the mother could also impact the function of the fetal/neonatal thyroid (4, 8, 10). Autoimmune hyperthyroidism also occurs in children born to mothers who were treated for GD years ago, but still have detectable circulating TRAb (4, 5, 11).

Fetal hyperthyroidism can cause goiter, heart failure with non-immune hydrops, advanced bone maturation, intrauterine growth retardation, preterm birth and even fetal death (1, 5, 6, 8). Therefore, a mother with a history of GD should be closely followed up during pregnancy. American Thyroid Association (2017) and European Thyroid Association (2018) guidelines have recommended taking measurements of maternal TRAb as soon as pregnancy is confirmed, and if elevated, repeating them at 18-22 weeks of gestation (12, 13). Neonatal autoimmune hyperthyroidism is usually transient and occurs in 1.5-2.5% of babies of GD mothers, but it is associated with an increased risk of long-term morbidity and mortality (1, 5, 14). Hyperthyroidism itself can lead to severe complications such as cardiac insufficiency, liver dysfunction, coagulopathy, craniostenosis, microcephaly and neurodevelopment disabilities (1, 14). Practice guidelines also include measurements of TRAb value in cord blood and serum levels of free thyroxine (fT4) and TSH between the 3rd and 5th day of life in neonates born to mothers with GD. Additionally, clinical observation in the first 2-3 months of life is recommended (1, 15, 16). Several studies have demonstrated that positive TRAb levels in cord blood correlate with the likelihood of development of hyperthyroidism in the first two weeks of life, whereas negative antibodies are associated with no risk of neonatal hyperthyroidism (1, 6, 14).

In fetuses/newborns of ATD treated mothers, the ATD passage across the placenta may increase the risk of the development of transient fetal/neonatal hypothyroidism (5, 15–17). It is usually observed in the first days of life until the ATD has been metabolized in the newborn’s body (9, 17, 18). Some newborns born to mothers with GD may also present central hypothyroidism (CeH) due to impaired regulation of the fetal hypothalamic-pituitary-thyroid (HPT) axis (19).

The aim of our observational study was to evaluate different types of thyroid dysfunction in babies with neonatal hyperthyroidism.

## Materials and Methods

This is an observational study including children hospitalized at the Department of Paediatrics and Endocrinology of the Medical University of Warsaw, Poland, between 2014 to 2021, due to neonatal hyperthyroidism. The study was approved by the Bioethics Committee at the Medical University of Warsaw. Medical records of 14 infants (10 boys, 4 girls) were analyzed. The following maternal data were collected: the history of mother’s thyroid disease, time of diagnosis (before or during pregnancy), TRAb levels and type of treatment (ATD, RAI therapy, thyroidectomy). In newborns the following data were analyzed: presence of obstetric ultrasound anomalies, gestational age, birth weight, birth length, birth head circumference, Apgar score, signs of hyperthyroidism. Birth growth parameters were reported in percentile ranks using Fenton 2013 Growth Calculator (https://peditools.org/fenton2013/) for each child. Infants were classed as small-for-gestational age (SGA) when their birth weight and/or length parameters were below the 10th percentile for gestational age (20). Time of TSH, fT4, free triiodothyronine (fT3) and TRAb levels normalization, the treatment modality was analyzed in each child.

Serum TSH (µIU/ml), fT4 (ng/dl) and fT3 (pg/ml) levels were measured by immunofluorescence method using the Architect i1000SR analyzer (Abbott Diagnostics, Abbott Park, Illinois, USA). The TRAb levels were measured by electrochemiluminescence immunoassay (ECLIA) with the Cobas e801 analyzer (Diagnostics Roche, Basel, Switzerland). Biochemical measurements were interpreted in relation to the reference range. Chosen anthropometric and biochemical data are also presented as means with standard deviation (SD) and minimum and maximum values.

## Results

The maternal, fetal and neonatal characteristics are presented in **Table 1**.

**Table 1:** The maternal, fetal and neonatal characteristics.

	Maternal thyroid disease history				Fetus	Delivery		Neonates			
	
Patient	Time of diagnosis	Treatment before pregnancy	Treatment during pregnancy	TRAb (IU/L) (>20 GW)	Symptoms of HT	GA(weeks)	Type of delivery	Apgar score	Weight gram (pc)	Length cm (pc)	Head cm (pc)
1	GD (*4yrs)	TD, L-T4	L-T4	na	no	33	CS (FF)	10	1800 (29)	47 (92)	33 (97)
2	GD (T1)		MMI	14.8	T,G	38	CS (GDM)	10	3530 (79)	55 (>99)	34 (52)
3	GD (*3mth)	ATD	PTU	9.23	no	38	CS	10	3600 (86)	52 (92)	35 (62)
4	GD (*)?		MMI+Encorton (from 28GW)	> 40	no	33	CS (FF)	10	2190 (67)	49 (99)	31 (69)
5	GD (*10yrs)	RI, L-T4	L-T4, MMI (from 22GW)	37	no	38	CS (ophthalmopathy)	10	2960 (42)	50 (72)	34 (62)
6	GD (T1)		PTU (T1), MMI (T2,3)	11	no	39	CS (FF)	10	4000 (91)	56 (99)	35 (63)
7	GD (*6yrs)	RI, L-T4	L-T4	na	no	39	CS (previous CS)	10	3760 (80)	57 (>99)	35 (63)
8	GD (T1)		no	3.34	no	39	CS (previous CS)	10	2970 (28)	53 (93)	32 (6)
9	GD (T1)		PTU (T1), MMI (T2,3)	31.2	no	41	CS (FF)	10	3850 (55)	58 (>99)	34 (13)
10	GD (T1)		no	2.51	no	39	CS (lack of progression)	10	3310 (46)	52 (78)	35 (63)
11	GD (*3mth)	ATD	PTU (T1), L-T4 (from T2)	na	no	38	VD	8-9-10	3580 (85)	56 (100)	43 (100)
12	GD (T1)		MMI, L-T4 (from T3)	6.8	no	41	CS (lack of progression)	10	3395 (34)	57 (99)	35 (44)
13	GD (*2mth)	ATD	MMI (to 23GW)	5.11	G	39	VD	10	2925 (16)	54 (95)	34 (36)
14	HD (*5yrs)	L-T4	L-T4 (T1)	14	no	36+6/7	VD	7-8-10	2940 (52)	50 (78)	34 (70)

GD, Graves’ disease; HD, Hashimoto disease; *, diagnosis before pregnancy; yrs, years; mth, months; T, trimester; TD, thyroidectomy; L-T4, levothyroxine; ATD, antithyroid drug; RI, ^131^ Iodine therapy; MMI, methimazole; PTU, propylothiouracyl; TRAb, thyrotropin receptor antibodies; GW, gestational weeks; na, not available; HT, hyperthyroidism; T, tachycardia; G, goiter; GA, gestational age; CS, caesarean section; VD, vaginal delivery; GDM, gestational diabetes mellitus; FF, fetal factors; pc, percentile; cm, centimeter.

Cut-off point for positive TRAb: >1.75 IU/L.

In our study 93% of infants (13 out of 14 cases) with neonatal hyperthyroidism were born to mothers with GD. Seven mothers were diagnosed before pregnancy (three of them were treated with ATD, two were after RAI therapy and one after thyroidectomy) and six mothers were diagnosed with GD in the first trimester of pregnancy. Only one neonate, presented as case 14, was born to a mother diagnosed five years before pregnancy with hypothyroidism in the course of Hashimoto thyroiditis and treated with levothyroxine (L-T4) until the end of the first trimester of pregnancy. The treatment was withdrawn because of TSH inhibition. Positive TRAb levels were detected in her after 20 weeks of gestation. From the second trimester she did not need L-T4 administration until the end of pregnancy and after delivery. In three cases maternal TRAb levels were not available for analysis, in all the other cases TRAb levels evaluated after 20 weeks of gestation were positive (ranging from 2.51 to more than 40 IU/L). Only in two cases (14%) the presence of fetal goiter and tachycardia was confirmed using ultrasound scans during pregnancy (cases 2 and 13). Majority of newborns (85%) were born in time; two babies were born preterm at 33rd week of gestation. All the babies were born in a good condition, none of them was born as SGA. In 79% of cases (11 out of 14) the delivery was by caesarean section.

### Groups of Patients Divided According to Thyroid Dysfunction After Birth

Characteristics of thyroid function and the type of therapy in all the studied babies are presented in **Table 2**.

**Table 2:** Characteristics of thyroid function and the type of therapy in all the studied babies.

	First thyroid blood test (start of treatment)					Thyroid blood test (end of MMI treatment)					Treatment		Follow-up	
Patient	Age	TSH µIU/ml	fT4 ng/dl (pmol/l*)	fT3 pg/ml (pmol/l*)	TRAB IU/L	Age	TSH µIU/ml	fT4 ng/dl (pmol/l*)	fT3 pg/ml	TRAB IU/L	Treatment duration (d)	IDD mg/kg b.w	Observation period	Treatment follow-up
1	6 d	**0.00**	76.03* (9-21)	29.52* (3.8-6.0)	na	28 d	**0.00**	1.33	4.0	**5.27**	MMI (6-28)	0.5	7 yrs	LT4 (2/12-6,5 yrs)
2	3/9 d	**0.138**/**0.02**	78.59* (13.9-26.1)/**3.6**	14.61* (3-8.1) /**11.04**	**6.26**	22 d	5.97	0.82	4.13	na	MMI (9-22)	0.5	3.5/12	–
3	3/5 d	**0.02/0.09**	27.05*/38.5* (10.94-23.68)	na/18.21* (3.44-7.59)	na	22 d	**0.28**	0.93	4.71	**2.21**	MMI (5-22)	0.5	3/12	–
4	5/7 d	**0.051/0.005**	1.76**/3.18**	**5.62/7.29**	**21.2**	30 d	1.27	**<0.42**	1.76	**8.05**	MMI (7-30)	0.6	9/11	LT4 (1/12-na)
5	7 d	**<0.01**	**2.36**	4.84	**16.4**	51 d	**0.51**	0.87	3.19	**3.23**	MMI (5-51)	0.3	2/12	–
6	3/7 d	2.588/**0.373**	34.48*/34.96* (9-52- 30.8)	4.99*/na* (3.44-7.59)	**8.16/7.36**	22 d	**19.93**	8.71* (11.1-27.3)	2.66	**3.93**	MMI (7-22)	0.5	9/12	LT4 (22d-continue)
7	4 d	2.143	**2.06**	na	**2.43**	14 d	na	na	na	0.96	Propranolol (4-14)	0.5	3/12	–
8	3 d	2.34	**2.67**	**6.18**	**4.76**	51 d	na	na	na	**1.85**	Propranolol (5-51)	0.5	4/12	–
9	5 d	1.74	**2.54**	na	**5.27**	34 d	9.26	**0.77**	3.38	**1.76**	MMI (5-34)	0.25	3/12	–
10	2/5 d	19.06/**1.1**	1.42/1.74	**5.68/**2.93	**2.3**	15 d	1.67	1.24	3.87	**2.3**	Propranolol (2-4)	0.5	15 d	–
11	2 d	**0.99**	1.17	2.74	< 0.8 CB	42 d	6.24	13.05* (10.29-21.88)	na	na	observation	–	1.5/12	–
12	3 d	6.54	**1.98**	na	**2.24** CB	3/12	**0.85**	1.02	3.98	na	observation	–	10/12	LT4 (<3/12-10/12)
13	25 d	**0.5**	1.22	4.09	**1.92**	3.5/12	**0.75**	1.12	3.72	1.34	observation	–	6.5/12	–
14	2 d	1.76	55.67* (13.9-26.1)	na	**5.76**	20 d	5.91	1.15	3.78	na	MMI (2-20)	0.4	6/12	–

TSH, thyroid-stimulating hormone; fT3, free triiodothyronine; fT4, free thyroxine; TRAb, thyrotropin receptor antibodies; MMI, methimazole; LT4, levothyroxine; d, day of life; yrs, years; na, not available; CB, cord blood; IDD, initial daily dose; b.w, body weight; Normal values: TSH - 0-3 days: 1.3-19.0 µIU/ml, 3-70 days: 0.6-17.0 µIU/ml, 70-356 days: 0.88-5.42 µIU/ml; fT4 - 0-3 days: 0.80-1.90 ng/dl, 3-70 days: 0.80- 1.70 ng/dl, 70-356 days: 0.89-1.72 ng/dl; fT3 - 0-12/12: 2.24-4.94 pg/ml; normal values of fT4 and fT3 in pmol/l are enclosed in bracket, next to the result; Cut-off point for positive TRAb: >1.75 IU/L.

#### 1 Overt Hyperthyroidism

Six out of the 14 babies (43%, cases: 1-6) showed overt hyperthyroidism with highly increased TRAb levels (mean 13.0 ± 7.0 IU/L, range: 6.26 - 21.2 IU/L). Maternal TRAb levels measured after 20 weeks of gestation were also high (mean 22.4 ± 14.8 IU/L, from 9.23 to more than 40 IU/L). All those babies required ATD treatment, which was started between 5 to 9 days of life with an initial daily dose of around 0.5 mg/kg. Mean duration of ATD treatment was 22 days (range: 13 - 46 days). Three babies in this group required further L-T4 supplementation (cases 4 and 6 subsequently to MMI therapy and case 1 one month after the end of MMI treatment). In two of them central hypothyroidism was diagnosed (cases 1 and 4) and in one primary hypothyroidism (case 6). One neonate (case 1) was born prematurely in the 33rd week of pregnancy. Only one child in these group (case 2) presented goiter and tachycardia in fetal life. Tachycardia was observed after the birth in all that babies.

#### 2 Hyperthyroidism With Low Normal TSH Level

Six out of 14 children (43%, cases: 7-10,12,14) presented hyperthyroidism with slightly increased fT4 and/or fT3 levels and TSH levels in the lower limit of the normal range coinciding with positive TRAb serum levels, but not exceed 6 IU/L (mean 3.8 ± 1.6 IU/L, range: 2.24 - 5.76 IU/L). Three of these babies due to tachycardia received only propranolol therapy for 2 to 46 days with an initial dose of 0.5 mg/kg. Two other children (cases 9 and 14) were treated for 29 and 18 days with initial dose of MMI 0.25 and 0.4 mg/kg, respectively. The neonate born to a mother diagnosed with Hashimoto thyroiditis before pregnancy (case 14) also demonstrated transient tachycardia. One neonate (case 12) presented a slightly elevated fT4 level with a normal TSH level in the third day of life, which did not require any pharmacological therapy. However, in the following few weeks decrease in TSH levels below the lower limit of the range and low normal fT4 levels were found and L-T4 supplementation was administered for seven months.

#### 3 Isolated TSH Suppression

Normal thyroid hormone levels with TSH levels below the lower limit of the range were observed in the neonates presented as cases 11 and 13. Those two babies did not require pharmacological therapy. In one of them (case 13) low TSH levels maintained until the fourth month of life and next spontaneously normalized. Maternal TRAb levels in those cases were 3.6 IU/L before pregnancy (not evaluated during pregnancy) in case 11 and 5.11 IU/L during pregnancy in case 13.

## Discussion

This study analyzed the thyroid function disturbances in infants diagnosed with hyperthyroidism in the neonatal period. As expected, almost all babies were born to mothers with GD, except for one child who was born to a mother with Hashimoto thyroiditis treated with L-T4. The presence of TRAb is rarely documented in previously hypothyroid individuals. In the above-mentioned case, after the first trimester of pregnancy the L-T4 supplementation had to be withdrawn because of TSH inhibition, but the mother in question did not develop overt hyperthyroidism and did not need to be treated with ATD. She remained euthyroid until the end of pregnancy and after delivery. A similar case was described by Kiefer et al. (21). The authors suggested that this phenomenon of unique coincidence of TSAb-induced fetal hyperthyroidism and maternal hypothyroidism results from the disappearance of the stimulating effect of TSAb on the mother’s thyroid because of severe chronic autoimmune-induced damage of thyroid tissue (21).

Typically, the presence of TRAb in the mother can lead to the development of autoimmune hyperthyroidism in the fetus and neonate (17, 22). It is recommended that the fetus should be closely monitored throughout pregnancy when maternal TRAb values exceed 5 IU/L or if TRAb levels are 3 times higher than the upper limit of the normal range (12, 13). The study by Gietka-Czernel et al. (4) showed that fetal goiter is the earliest and most characteristic sign of hyperthyroidism. Tachycardia and advanced bone age occur later. In the present study, the presence of fetal goiter was documented in two babies with maternal TRAb levels of 14.8 IU/L and 5.11 IU/L, respectively. Surprisingly, one child (case 4), that of the mother with TRAb levels higher than 40 IU/L during pregnancy, did not have any symptoms of thyrotoxicosis. This baby was born preterm and developed overt hyperthyroidism, followed by CeH diagnosed soon after withdrawal of MMI therapy. His mother had poorly controlled GD with thyroid crisis in the 28th week of gestation. We suppose that CeH in this child resulted from the coincidence of high dose of MMI, devastating biosynthesis of thyroid hormones in the fetus, and the impact of high TRAb levels on the ultra-short loop pituitary feedback mechanism during fetal development.

In order to reach fetal euthyroid status when the mother is treated with ATD, the European Thyroid Association and American Thyroid Association guidelines recommend the use in mothers of the lowest possible effective ATD dose which maintains serum fT4 levels at or slightly above the upper limit of the pregnancy-specific ranges (12, 13). Iwaki et al. (23) investigated the dose-dependent effect of ATD on both maternal and fetal thyroid hormone status and confirmed a dose-dependent influence of ATD on the difference in serum fT4 levels between mothers treated with high propylthiouracil dosage (>100 mg daily) or MMI dosage (>5 mg daily) and their neonates, who had significantly lower cord blood fT4 levels than maternal serum fT4.

Our study indicates that tachycardia could be the sole clinical manifestation of hyperthyroidism in a newborn. Other symptoms, such as poor weight gain despite good appetite, irritability, hypertension, tachypnoe and ocular protrusion, have been reported in other studies (11, 24, 25).

Our observations confirmed that the types of thyroid dysfunctions in neonates are mainly determined by the TRAb level in the mother during pregnancy. The multicenter study by Banigé et al. (17) indicates that the optimal cut-off value of maternal TRAb is 2.5 IU/L for predicting fetal thyroid hypertrophy and 5.9 IU/L for predicting neonatal thyroid dysfunction. When using the neonatal TRAb level, measured in cord blood at delivery or in peripheral blood between 0 and 5 days of life, the recommended cut-off value for predicting thyroid dysfunction in a neonate is 6.8 IU/L with a sensitivity of 100% and a specificity of 94% (17). Our observations are in line with the above-mentioned results except for the risk of thyroid hypertrophy. The coincidence of high maternal TRAb levels with normal thyroid function in a neonate born to a mother with a history of GD is rarely described (4, 18, 26). It can be explained by the balance of simultaneously maternally transmitted TSAb and TBAb levels in the neonate’s circulation. An analysis by Benlarbi et al. (26) shows that the majority of neonates with TRAb levels above 6 IU/L develop transient hyperthyroidism, 15% are diagnosed with primary hypothyroidism or CeH, and only 12% remain euthyroid. In addition, it has been confirmed that babies born to mothers after definitive treatment of GD before pregnancy are also at risk of hyperthyroidism at birth. Shortly after RAI therapy, TRAb levels increase. The study by Yoshihara et al. (10) strictly indicates that the risk of autoimmune hyperthyroidism in newborns is inversely related to the time lapse after RAI therapy in the mother. On the other hand, in women who underwent RAI therapy several years before pregnancy and have only slightly elevated TRAb levels in early pregnancy, TRAb levels could rise to high values at delivery (27). In our study, two mothers underwent RAI therapy three years before pregnancy. One of them developed severe thyrotoxicosis with high TRAb levels (37 IU/L) and ophthalmopathy during pregnancy. The risk of fetal/neonatal hyperthyroidism is lower in babies born to women with GD treated with thyroidectomy before pregnancy, but TRAb levels should also be assessed in them early in pregnancy and at 18-22 weeks of gestation (12, 13).

Neonatal hyperthyroidism is not the only consequence of GD in the mother. The non-obvious consequence of maternal GD leading to prolonged CeH should also be taken into account. It has been confirmed that increased transplacental passage of maternal thyroid hormones may disturb physiologic maturation and regulation of the fetal HPT axis during intrauterine life (19). Excessive fetal thyroid hormones production in response to stimulation by maternal TRAb may also diminish fetal TSH secretion. Overexposure to thyroid hormones might alter the fetal pituitary TSH secretion set point (28, 29). Animal studies have shown that increased levels of thyroid hormones *in utero* could decrease the number of fetal pituitary thyrotrophs and TSH receptors (30, 31). Neonatal pituitary hyporesponsiveness to thyrotropin-releasing hormone (TRH) stimulation and to fT4 levels is also well-documented (29, 32). CeH is usually transient and appears at birth or follows transient neonatal thyrotoxicosis after a decrease in TSAb activity (33). The return of the HPT axis to normal function usually takes from 3 to 19 months, but in some cases as long as 3.5 years (1, 28, 29, 32). In our group in one patient the process lasted as long as 6.5 years. The much longer persistence of CeH than that of detectable TRAb levels suggests that it is related not only to the effect of TRAb on the ultra-short loop axis, but it is also associated with marked HPT axis impairment including receptors sensitivity and gene expression during fetal life. Disclosure of CeH could be delayed due to its masking by transient hyperthyroidism observed in the first weeks of life, therefore there is a need for prolonged monitoring of thyroid function in the offspring of mothers with GD.

The main limitation of the present analysis is the small size of the study group. On the other hand, we analyzed a selected group of children with thyroid dysfunction, i.e., only those patients who required hospitalization.

## Conclusion

Our study highlights the need for prolonged monitoring of thyroid function in children born to mothers with GD. Transient hyperthyroidism is the main thyroid dysfunction in that group of children, but primary or central hypothyroidism requiring L-T4 supplementation could appear at birth or after withdrawal of ATD treatment. Diagnosis of CeH could be delayed due to its masking by transient hyperthyroidism. Prolonged TSH suppression after TRAb elimination should be considered as a signal announcing CeH.

## Data Availability Statement

The raw data supporting the conclusions of this article will be made available by the authors, without undue reservation.

## Ethics Statement

The studies involving human participants were reviewed and approved by Medical University of Warsaw. Written informed consent from the participants’ legal guardian/next of kin was not required to participate in this study in accordance with the national legislation and the institutional requirements.

## Author Contributions

BP analyzed maternal and infants data, wrote the manuscript, and collected the literature data. MR designed the study, recorded and analyzed maternal and infants data, wrote the manuscript and prepared tables, collected the literature data. EW-S designed the study, recorded and analyzed maternal and infants data, wrote the manuscript, collected the literature data. AK recorded and analyzed maternal and infants data and wrote the manuscript. All authors contributed to the article and approved the submitted version.

## Conflict of Interest

The authors declare that the research was conducted in the absence of any commercial or financial relationships that could be construed as a potential conflict of interest.

## Publisher’s Note

All claims expressed in this article are solely those of the authors and do not necessarily represent those of their affiliated organizations, or those of the publisher, the editors and the reviewers. Any product that may be evaluated in this article, or claim that may be made by its manufacturer, is not guaranteed or endorsed by the publisher.
